# Acute Treatment with a Novel TRPC4/C5 Channel Inhibitor Produces Antidepressant and Anxiolytic-Like Effects in Mice

**DOI:** 10.1371/journal.pone.0136255

**Published:** 2015-08-28

**Authors:** Li-Ping Yang, Fang-Jie Jiang, Gui-Sheng Wu, Ke Deng, Meng Wen, Xiaoju Zhou, Xuechuan Hong, Michael X. Zhu, Huai-Rong Luo

**Affiliations:** 1 State Key Laboratory of Phytochemistry and Plant Resources in West China, Kunming Institute of Botany, Chinese Academy of Sciences, Kunming, Yunnan 650201, China; 2 University of Chinese Academy of Sciences, Beijing 100039, China; 3 Yunnan Institute of Traditional Chinese Medicine and Materia Medica, Kunming, Yunnan 650223, China; 4 State Key Laboratory of Virology, Key Laboratory of Combinatorial Biosynthesis and Drug Discovery (Wuhan University), Ministry of Education, Wuhan University School of Pharmaceutical Sciences, Wuhan 430071, China; 5 Department of Integrative Biology and Pharmacology, University of Texas Health Science Center at Houston, Houston, TX, 77030, United States of America; Chiba University Center for Forensic Mental Health, JAPAN

## Abstract

Transient receptor potential canonical (TRPC) channels are widely expressed in brain and involved in various aspects of brain function. Both TRPC4 and TRPC5 have been implicated in innate fear function, which represents a key response to environmental stress. However, to what extent the TRPC4/C5 channels are involved in psychiatric disorders remains unexplored. Here, we tested the antidepressant and anxiolytic-like effects of a newly identified TRPC4/C5 inhibitor, M084. We show that a single intraperitoneal administration of M084 at 10 mg/kg body weight to C57BL/6 male mice significantly shortened the immobility time in forced swim test and tail suspension test within as short as 2 hours. The M084-treated mice spent more time exploring in illuminated and open areas in light/dark transition test and elevated plus maze test. In mice subjected to chronic unpredictable stress, M084 treatment reversed the enhanced immobility time in forced swim test and decreased the latency to feed in novelty suppressed feeding test. The treatment of M084 increased BDNF expression in both mRNA and protein levels, as well as phosphorylation levels of AKT and ERK, in prefrontal cortex. Our results indicate that M084 exerts rapid antidepressant and anxiolytic-like effects at least in part by acting on BDNF and its downstream signaling. We propose M084 as a lead compound for further druggability research.

## Introduction

Depression is a devastating psychiatric disorder that severely affects the quality of life of both patients and their family. The prevalence of depression is widespread among world populations, making it one of the leading causes to the global disability and socioeconomic burden [1–3]. In the etiology and pathophysiology of depressive disorders, chronic stress is one of the most important contributing factors [4]. This explains the strong comorbidity between depression and anxiety [5] and the similar effectiveness of pharmacological therapies for both disorders [6].

The first-line treatments for depressive disorders are antidepressant drugs developed based on the monoamine-deficiency hypothesis. These drugs cause a quick increase in the monoamine levels of brain, but they exhibit a long latency to relieve the symptoms. In addition, only one third of the major depressive disorder (MDD) patients receiving antidepressants achieve complete remission following a single treatment, while up to one-third of the patients fail to remiss even after consecutive treatments, constituting the so called “treatment-resistant depression” (TRD) [7]. Alternative treatments, such as deep brain stimulation (DBS), electroconvulsive therapy (ECT), and repetitive transcranial magnetic stimulation (rTMS), are also only effective for certain types of these disorders. Technical limitations of these methods also further restrict their clinical applications. Thus, developing cost-effective pharmacotherapies remains to be an important approach to mitigate the suffering and burden of depressive disorders.

Transient receptor potential canonical (TRPC) channels make up a subfamily of calcium-permeable nonselective cation channels, which are implicated in neural development, brain function, and neurological disease [8]. There are seven TRPC channels in mammalian species. Among them, TRPC2 is a pseudogene in humans. The remaining members of the TRPC subfamily are classified into three groups according to sequence homology, TRPC1, TRPC3/C6/C7, and TRPC4/C5. Among them, TRPC1 is known to form heteromeric channels with not only other TRPC members, especially TRPC4/C5, but also members of other TRP subfamilies [9–11]. TRPC channels could be activated by G_αq/11_-coupled receptors and tyrosine kinase-linked receptors through phospholipase C activation or diacylglycerol production [8]. Functionally, TRPC1 has been implicated in the control of neural development and axon guidance [8]. While TRPC4 and C5 channels were reported to be involved in various physiological and pathophysiological processes, such as vascular smooth muscle, endothelial function, adiponectin regulation, and oxidative stress [12–14]. The protein kinase calcium/calmodulin-dependent kinase II beta (CaMKIIβ) was found to be a critical effector of TRPC5 function in neurons [15–19]. TRPC5 has been reported to negatively regulate neurite outgrowth, dendrite morphogenesis and connectivity. The role of TRPC4 in axonal regeneration and epileptiform burst firing were also reported [20, 21]. Both TRPC4 and TRPC5 have been shown to be involved in anxiety-like behavior in the mouse model of fear conditioning test [22, 23]. Studies also suggest that TRPCs may be targeted for therapeutic treatment of neurological diseases [8]. Recently, a new class of TRPC4/C5 inhibitors was identified using a cell-based high throughput screening assay [24, 25]. The lead compound, M084, exhibited very good selectivity on TRPC4, TRPC5, and TRPC1/C4 channels as compared to several other TRP channels as well as voltage-gated Ca^2+^, Na^+^ and K^+^ channels tested [25]. In the present study, we investigated whether M084 has antidepressant and anti-anxiety effects. Our results show remarkable beneficial effects by a single treatment of mice with M084 for as short as 2 hours in multiple depression/anxiety-related behavioral tests both under normal conditions and after long-term chronic unpredictable stress (CUS) exposure. Accompanied with the antidepressant-like and anxiolytic-like effects and similar to other antidepressants, the M084 treatment caused increased signaling by brain-derived neurotrophic factor (BDNF) in prefrontal cortex (PFC). These results highlight TRPC4 and TRPC5 as new potential targets for the treatment of mood-related disorders.

## Materials and Methods

### Animals

C57BL/6 male mice (6 weeks, 18–22 g) were purchased from Institute of Laboratory Animal Sciences (Chengdu, Sichuan, China). The animals were housed at 4 per cage under specific pathogen free conditions of 12/12 hours light/dark cycles, temperature of 22 ± 2°C and humidity of 50–60%, with free access to food and water. They were habituated to the animal facility for one week prior to behavioral testing. Twelve mice per group were used in behavioral studies and six mice per group for gene expression experiment. The experiments were performed in strict accordance with the protocols approved by the Institutional Animal Care and Use Committee of the Kunming Institute of Botany, Chinese Academy of Sciences. All experiments were carried out in accordance with the ARRIVE guidelines (S1 ARRIVE Guidelines Checklist).

### Drugs and treatment

**Fig 1:**
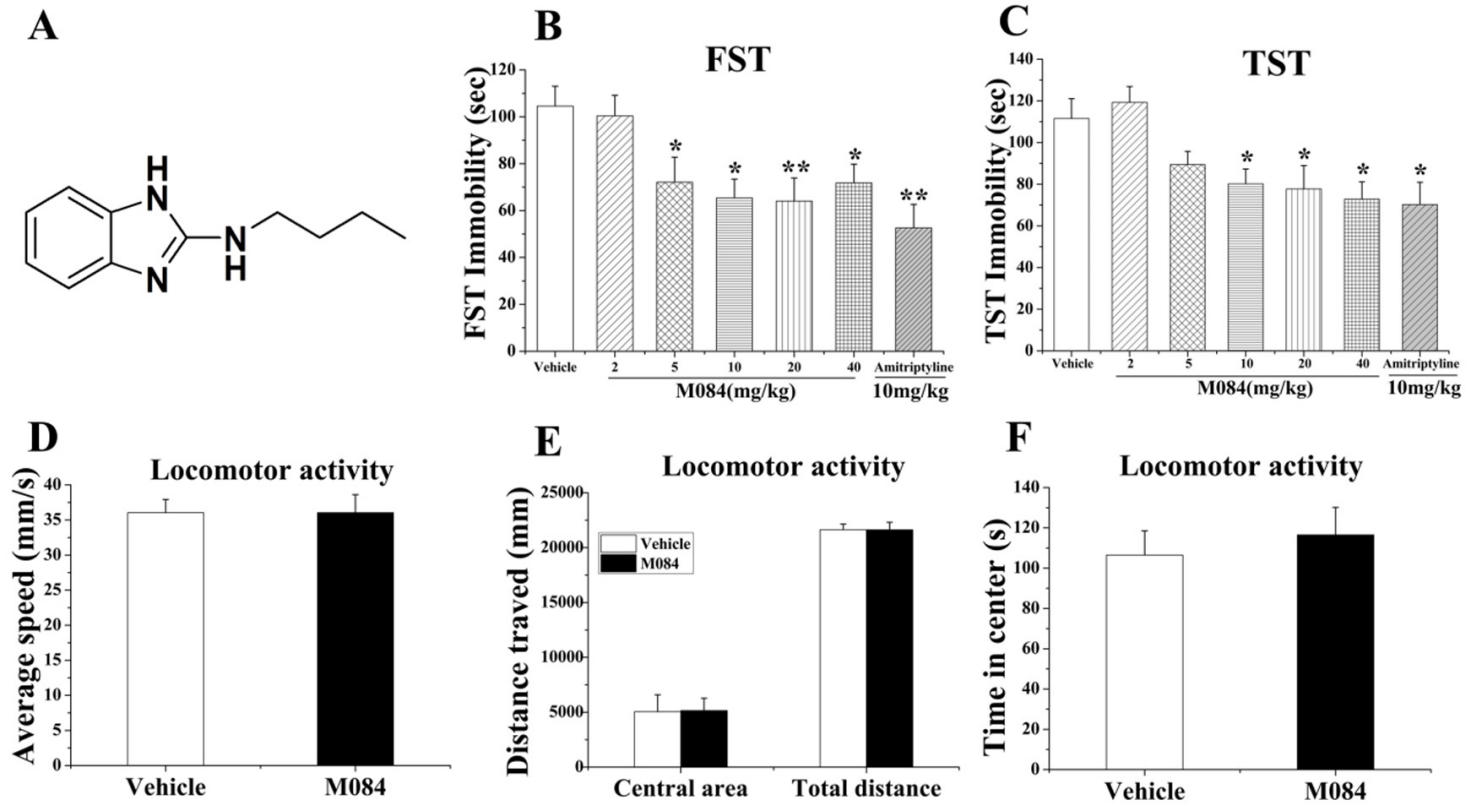
M084 exhibits antidepressant-like effects in mice. (A) The chemical structure of M084. (B&C) M084 reduced immobility time in forced swim test (FST, B) and tail suspension test (TST, C). Amitriptyline, a known antidepressant, was used as a positive control (10 mg/kg, 2 hrs). (D-F) In locomotor activity test, mice treated with vehicle and M084 (10 mg/kg, 2 hrs) showed no difference over the average speed (D), distance in central area and total distance traveled (E), and time in central area (F). Results are presented as mean ± SEM. N = 10–12/group, * *p* < 0.05, ** *p* < 0.01.

The compound M084, n-butyl-1h-benzimidazol-2-amine (Fig 1A), was synthesized as described [24] and dissolved in a water solution containing 10% of DMSO. The antidepressant amitriptyline and antianxiety drug diazepam (Sigma Aldrich, St Louis, MO, USA) were also dissolved in the water solution containing 10% of DMSO. Animals received intraperitoneal (*i*.*p*.) injection of either the solvent control (vehicle), or M084 (at 2, 5, 10, 20, and 40 mg/kg body weight) or amitriptyline (at 10 mg/kg body weight) or diazepam (at 1.5 mg/kg body weight) two hours before the behavioral tests. The dose of M084 was chosen based on the IC_50_ of M084 in cellular assays. The IC_50_ of M084 was 3.7 ~ 10.3 μM, for TRPC4βchannel by various assays in different cellular models and 8.2 ± 0.7 μM for TRPC5 by the FMP assay using DAMGO to stimulate Gi/o via the co-expressed μ receptor [25]. For the density of mice was close to water (mice were little heavier than water), the dose of 10 mg/kg was roughly equal to dose of 50 μM in mice. The concentration of M084 in brain of mice was about 5.8 μM, which was close to the IC_50_ of M084 in cellular assays. The dose of amitriptyline and diazepam were chosen based on the previous studies [26, 27].

### Behavioral studies

Mice were subjected to locomotor activity test first, followed by light-dark transition (L/D) test, elevated-plus maze (EPM), finally forced swim test (FST) or tail suspension test (TST). Different mice were used in TST and FST. CUS mice were subjected to locomotor activity test, novelty suppressed feeding test (NSFT), and FST, respectively.

Locomotor activity—Locomotor activity was evaluated to ensure that changes in behavioral tests are due to antidepressant responses rather than indirect effects of the drug on the locomotor activity. Mouse was placed in an open field chamber (Jiliang Software Technology, Shanghai, China) and its general locomotor behavior recorded using a video camera. After an acclimatization period of 3 min, the next 10 min of the locomotor activity was analyzed. The floor of the open field apparatus was cleaned with 70% ethanol between tests.

Forced swim test (FST)—FST was conducted as previously described [28]. Mice were individually placed in glass cylinders (diameter and depth 10 x 25 cm) with water (25 ± 1°C) filled up to 18 cm and allowed to swim for 6 min. The behaviors were recorded using the activity monitoring software (Jiliang Software Technology, Shanghai, China) for subsequent analysis. Duration of immobility is defined as the time the mouse spent floating in the water without struggling and making only those movements necessary to keep its head above the water. After the test, the mouse was removed from the tank, dried thoroughly with a towel, and returned to its home cage. The tank water was replaced after the swimming session of each mouse. The total duration of immobility was measured during the final 4 min of the 6-min test session.

Tail suspension test (TST)—TST was performed as previously described [29]. Briefly, an adhesive tape was wrapped around the mouse tail 1 cm from the tip end and tied together with the tail to a hook hung in the center from the ceiling of the observation chamber. The behaviors were recorded by the activity monitoring software described above. The mouse was considered immobile when it hung there without any attempt to struggle. The total duration of immobility during the last 4 min of a 6-min test session was determined for each mouse.

Elevated-plus maze (EPM)—The EPM apparatus (Jiliang Software Technology, Shanghai, China) consists of a black Plexiglas maze (40 cm above ground) with four arms (10 x 45 cm) and an intersection (10 x 10 cm). Two arms are open and the other two are enclosed. The test mouse was placed in the central zone, facing always the same closed arm, and allowed to move freely around the maze for 10 min. The session was recorded by a video camera and numbers of entries to the open and closed arms were quantified. After each trial, the boxes were cleaned with 70% ethanol and dried using paper towels.

Light-dark transition (L/D) test—The L/D test apparatus comprises of a box divided into two equal-sized compartments (Jiliang Software Technology, Shanghai, China). The light chamber is painted white and illuminated while the dark chamber is painted black and enclosed under a dark cover. The test mouse was placed in the dark box and the number of entries it made and the time it spent in the light box were recorded for 10 min using a video camera. After each trial the boxes were cleaned with 70% ethanol and dried using paper towels.

Novelty suppressed feeding test (NSFT)—Mice were food-deprived overnight before the test. Food pellets were placed in the center of an open field illuminated with light. The test mouse was placed in a corner of the arena and allowed to explore the open field for 10 min or when the mouse approached and took the first bite of the chow, whichever came first. The amount of food consumed in the home cage was measured right after the test as a control.

### Chronic unpredictable stress (CUS) procedures

The CUS procedures were performed as previously described [30]. All animals were matched with age and weight before commencement of CUS procedures. Mice were exposed to a variable sequence of unpredictable stressors for a period of 30 days. These stressors included cold stress (4°C, 10 min), cage rotation (1 hr), isolation overnight, food or water deprivation overnight, light on overnight, 45° tilted cage overnight, wet bedding overnight, 15 min inescapable foot shocks (0.7 mA intensity, 3-s duration with 1-s interval), restraint (2 hrs), and noise overnight. All stressors were randomly interspersed twice throughout the stress period. Behavioral tests were performed 12 hours after the CUS procedure was finished.

### Quantitative polymerase chain reaction (qPCR)

Mice were sacrificed right after behavioral tests by cervical dislocation and brain tissues dissected on ice. Total RNA was extracted from hippocampus and prefrontal cortex (PFC) using RNAiso plus (Takara) according to the manufacturer’s protocol. Reverse transcription was performed to obtain the cDNA by the Transcription Kit (Applied Biosystems) using oligo-dT primers. QPCR was carried out using Power SYBR Green PCR Master Mix (Applied Biosystems) and ABI 7500 system. The relative expression levels were analyzed using the 2^−ΔΔCt^ method and normalized to GAPDH. Primer sequences were: BDNF, forward 5’-TCATACTTCGGTTGCATGA AGG-3’, reverse 5’- AGACCTCTCGAACCTGCCC-3’; c-fos, forward 5’- CCG ACTCCTTCTCCAGCAT-3’, reverse 5’-TCACCGTGGGGATAAAGTTG-3’; GAPDH, forward 5’- CCAAAAGGGTCATCATCTCC-3’, reverse 5’-GAGGGGCC ATCCACAGTCTT-3’.

### Western blotting

Mice PFC were homogenized in protein lysis buffer containing 50 mM Tris-HCl, 150 mM NaCl, 1% Triton X-100, 1% sodium deoxycholate, 0.1% SDS, and 1% protease inhibitor cocktail (Roche Molecular Biochemicals, Nutley, NJ, USA). Proteins were separated on 10% SDS-PAGE gels and transferred onto PVDF membranes (Millipore, Billerica, MA, USA). Blots were incubated in primary antibodies overnight at 4°C. The following primary antibodies were used: mature BDNF (Proteintech, 1:100), ERK and pERK (Cell Signaling Technology, 1:1000), AKT and pAKT (Cell Signaling Technology, 1:1000), GAPDH (Proteintech, 1:2000). The next day, blots were washed three times and incubated with horseradish peroxidase (HRP)-conjugated secondary antibodies (Proteintech, 1:3000) for one hour. The blots were detected by the enhanced chemiluminescence technique (Millipore, Billerica, MA, USA) and the results analyzed using Gel pro 3.2 (Media Cybernetics, Silver Spring, MD, USA). Protein levels were normalized to GAPDH levels and phosphoproteins were normalized to the total proteins and expressed as the percentage of the same protein found in control animals.

### Distribution of M084 following intraperitoneal injection

Male C57BL/6 mice (8 weeks, 18–22 g) were anesthetized two hours after *i*.*p*. injection of vehicle or M084 (10 mg/kg). Then, serum, cerebrospinal fluid (CSF) and brain tissue were collected. Mouse brain was weighed and homogenized in equal volume of methanol. CSF and serum were dispersed into a polypropylene tube and mixed with equal volume and two times volume of methanol, respectively. The mixtures of serum, brain and CSF were centrifuged at 12,000 rpm for 15 min respectively. The supernatants were collected and filtered by a filter (0.2 μm, Agilent Technologies, Germany) for measurement.

The filtered supernatants were subjected to separation in an Acquity UPLC system (Waters, UK) equipped with an Acquity UPLC BEH C18 column (1.7 μm, 2.1 EH mm, Waters, UK). Mobile phases A and B consisted of 0.1% formic acid in water and acetonitrile, respectively. The flow rate was set at 0.2 mL/min. A 7.5-min elution gradient was performed as follows: (A: B), 40: 60 for 5 min after injection, 20: 80 at 5 min, 40: 60 at 5.1 min and up to 7.5 min. The MS analysis was performed by a Waters Xevo TQ-S mass spectrometer (Waters, UK) equipped with an ESI source in the positive-ion mode. A capillary voltage of 2 kV, a source temperature of 150°C and a desolvation temperature of 350°C were used. Desolvation and the cone gas flow were set as 800 L/h and 150 L/h, respectively, and the collision gas was 2 mL/min. The brain and serum sample were diluted by 100 times and the CSF sample was diluted by 50 times, respectively. A linear calibration curve was established with five concentration standards of M084 and plotted using the ratio of analyte peak area over IS peak area after integration by Masslynx 4.1 software (Waters Corporation). Retention time for M084 was 7.5 min. The concentration of M084 (C_brain/serum/CSF_) is calculated using the measured concentration (C_m_), the times of dilution during measured (100 or 50 times), and the times of dilution during sample preparation (2 times), as following: C_serum_ = C_m_× 100 × 3, C_brain_ = C_m_× 100 × 2, C_CSF_ = C_m_× 50×2.

### Statistical analysis

All data are expressed as means ± S.E.M. Statistical analysis was performed using two-tailed Student’s *t*-test or one-way analysis of variance (ANOVA) (SPSS version 13.0, Chicago, CA, USA), the Fisher least significant difference (LSD) test was employed for post hoc analyses. Probability value of *p* < 0.05 was considered as statistically significant.

## Results

### Acute treatment of M084 exhibits antidepressant-like and anxiolytic-like effects

The forced swim test (FST) and tail suspension test (TST) are commonly used to assess the behavioral despair and screen for antidepressants [31, 32]. Since TRPC channels have been implicated in anxiety-like behaviors [19, 33], we tested the antidepressant potential of the newly identified TRPC4/C5 inhibitor, M084, on mice. As shown in Fig 1, acute treatment with M084 (2, 5, 10, 20, or 40 mg/kg, *i*.*p*.) significantly reduced the immobility time of mice in FST [one-way ANOVA, F (6, 77) = 4.172, *p* = 0.001; Fig 1B], except at the dose of 2 mg/kg. Similarly, acute treatment with M084 at 10, 20 and 40 mg/kg, but not at 2 and 5 mg/kg, also significantly reduced the immobility time in TST [one-way ANOVA, F (6, 77) = 2.689, *p* = 0.020; Fig 1C]. The antidepressant-like effect is similar extents as the known antidepressant, amitriptyline. The administration of M084 (10 mg/kg, *i*.*p*.) worked best and did not alter the locomotor activities of the mice [t-test, *p* > 0.05; Fig 1D–1F].

**Fig 2:**
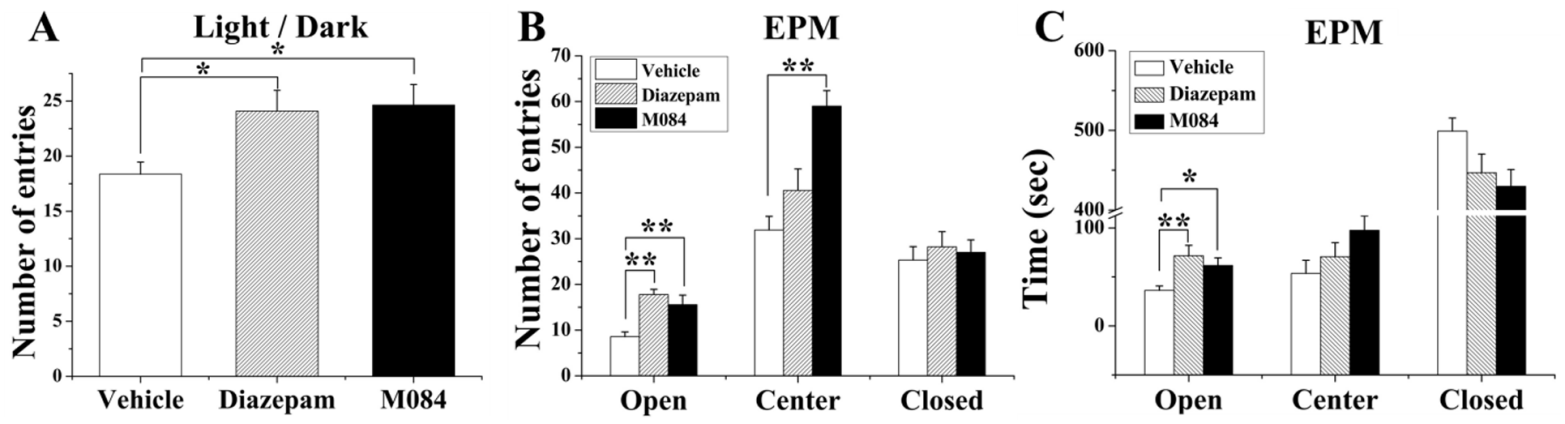
M084 exhibits anxiolytic-like effects in mice. (A) In the light/dark transition test, M084 treatment (10 mg/kg, 2 hrs) resulted in an increase in the number of entries in the light chamber. (B&C) In elevated plus maze (EPM) test, animals treated with M084 had more entries in all areas (B) and spent more time in the open arms and central area and less time in the closed arms (C) as compared to those treated with vehicle. Diazepam, a known antianxiety agent, was used as a positive control (1.5 mg/kg, 2 hrs). Results are shown as mean ± SEM. N = 12/group, * *p* < 0.05, ** *p* < 0.01.

To examine the possible anxiolytic-like effect of the TRPC4/C5 inhibitor, we subjected the mice to light/dark transition (L/D) test and elevated plus maze (EPM) test. In the L/D test, the M084-treated mice (10 mg/kg, *i*.*p*.) displayed increased time of entry into the light box compared to the vehicle-treated controls [one-way ANOVA, F (2, 30) = 4.398, *p* = 0.021; Fig 2A]. Similarly, in the EPM test, the M084-treated mice spent more time exploring by entering both open and the center of the maze more times [one-way ANOVA, open arms: F (2, 24) = 10.253, *p* = 0.001; central area: F (2, 24) = 13.467, *p* = 0.0001; Fig 2B] and cumulatively stayed significantly more time in open arms and the central area but less time in closed arms [one-way ANOVA, open arms: F (2, 24) = 5.196, *p* = 0.013; Fig 2C]. We also explored the effect of M084 at 2, 5, 20, and 40 mg/kg dosage. However, M084 had no effect on both light/dark test and EPM test at these dosages (data not shown). The above tests indicate that M084 exhibits antidepressant and anxiolytic-like effects at 10 mg/kg dosage in mice.

### M084 could cross the blood-brain barrier

**Fig 3:**
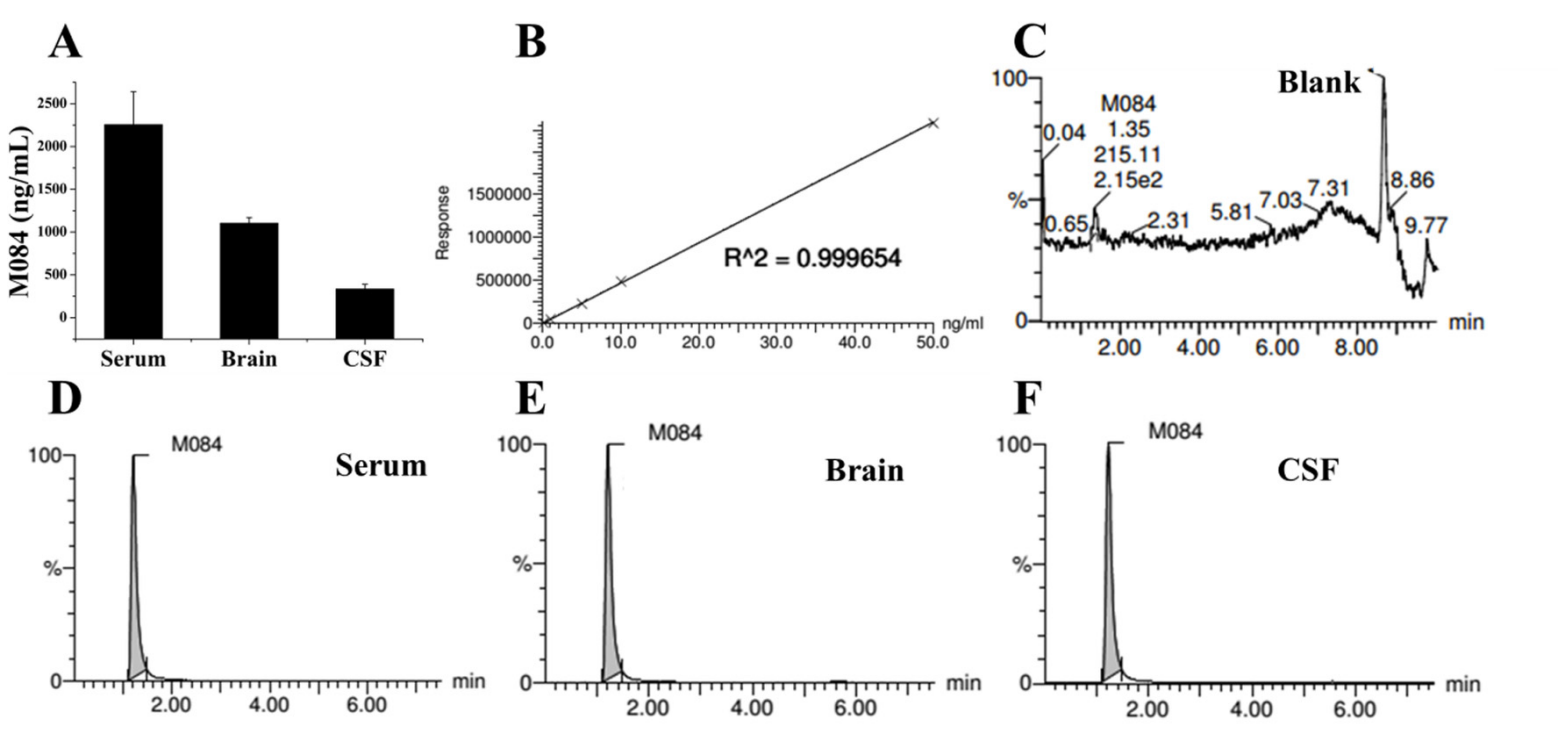
Distribution of M084 in serum, brain and CSF of mice. Serum, brain, and CSF samples were collected at 2 hours after treatment of M084 or vehicle. Concentrations of M084 were determined with LC-MS/MS analysis. (A) M084 can efficiently cross the blood-brain barrier at pharmacologically relevant doses (10 mg/kg, 2 hrs). Results are shown as mean ± SEM. (B) M084 calibration curve. Correlation coefficient = 0.999654, weighting factor = 1/x. (C-F) Representative LC–MS/MS chromatogram of blank brain sample, serum, brain and CSF, respectively.

In order to confirm if M084 exist in the brain after single dose of intraperitoneal injection (10 mg/kg, 2 hrs), the concentrations of M084 in the serum, brain and CSF were measured. The results show that the concentration of M084 is 2256.00 ± 384.00 ng/mL, 1105.00 ± 65.00 ng/mL, and 335.00 ± 55.00 ng/mL in serum, brain, and CSF, respectively (Fig 3). Thus, the concentration of M084 in brain of mice was about 5.8 μM, which was close to the IC_50_ of M084 in cellular assays. This result suggested that M084 could cross the blood-brain barrier to do its work at pharmacologically relevant doses.

### M084 exhibits antidepressant-like and anxiolytic-like effects in stressed mice

**Fig 4:**
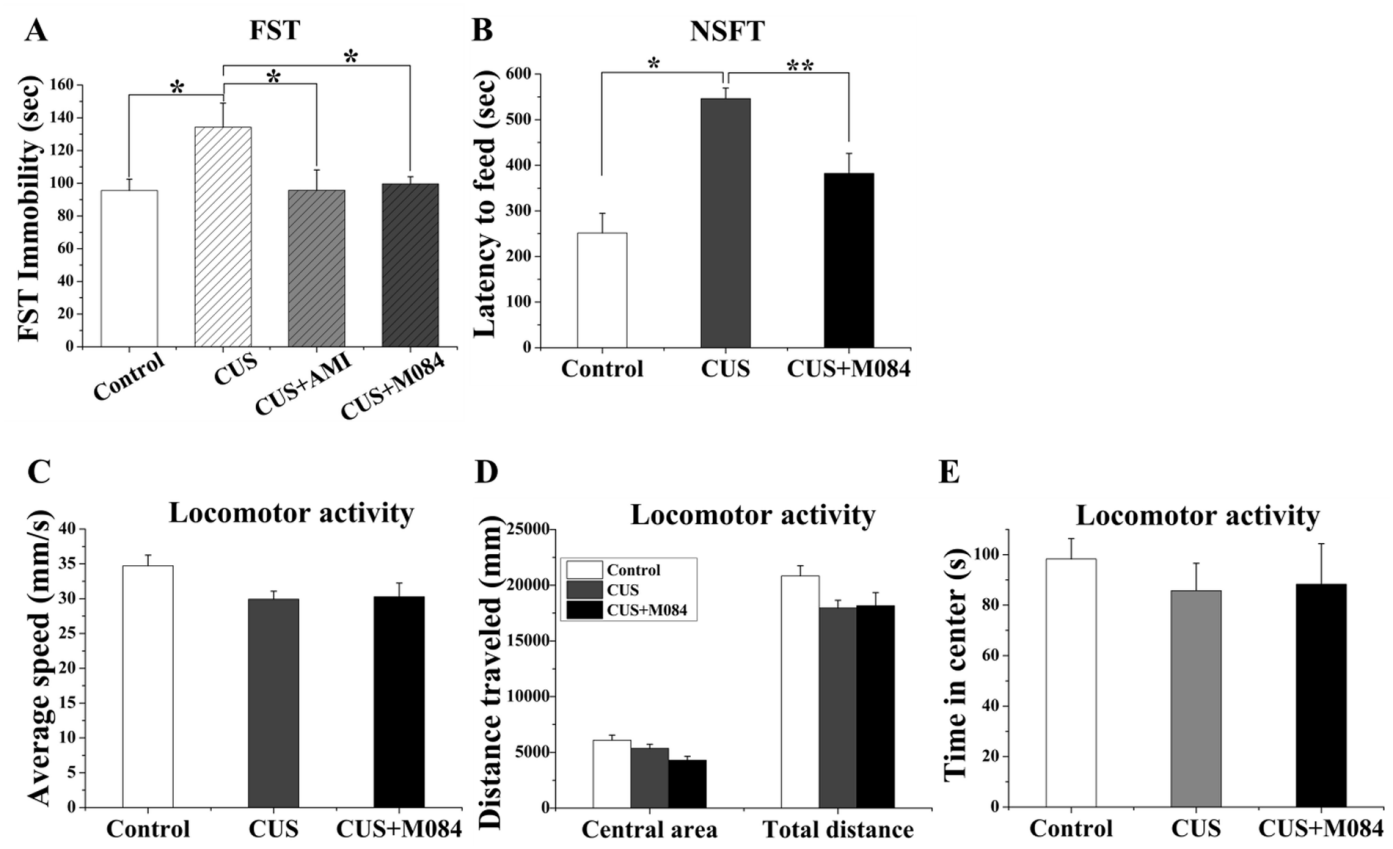
M084 treatment reverses the CUS-induced pathological behaviors. (A) M084 (10 mg/kg, 2 hrs) and amitriptyline (10 mg/kg, 7 days) treatment decreased immobility time in the FST of CUS-exposed mice. (B) In novelty suppressed feeding test (NSFT), CUS increased the latency to feed, while M084 treatment reversed such an effect. (C-E) In locomotor activity test, mice subjected CUS displayed moderate changes over the average speed (C), distance traveled in central area and the total distance traveled (D), and time in central area (E). M084 treatment had no effect on these changes induced by CUS. Results are showed as mean ± SEM. N = 12/group, * *p* < 0.05, ** *p* < 0.01, *** *p* < 0.001.

To further validate the effects of M084 on suppressing depression and anxiety, we used the chronic unpredictable mild stress (CUS) paradigm, which is considered to be one of the paradigms most relevant to etiological and behavioral changes that are clinically observed in patients with depressive disorders [34]. As expected, mice subjected to CUS displayed a significant increase in immobility time in the FST test, the treatment of M084 abolished the effect of CUS on immobility time similar to amitriptyline [one-way ANOVA, F (3, 44) = 3.413, *p* = 0.027; Fig 4A].

Novelty-suppressed feeding test (NSFT) is an effective paradigm for assessing the anxiolytic and chronic antidepressant efficacy of a drug [35]. In the NSFT, mice subjected to CUS exhibited a significant increase in the latency to feed in a novel environment, an indication of elevated anxiety levels. Again, the treatment of M084 reversed the effect of CUS on the latency to feed in the NSFT [one-way ANOVA, F (2, 33) = 5.203, *p* = 0.012; Fig 4B], demonstrating its anxiolytic-like effect in the CUS model. The treatment with M084, however, did not alter home cage feeding conducted immediately following the NSFT (data not shown), indicating that the effect of M084 was not due to a general increase in feeding. Furthermore, although CUS also led to reduced locomotor activities in mice the administration of M084 was ineffective at these activities (Fig 4C–4E). Therefore, the observed antidepressant and anxiolytic-like effects of M084 in the CUS model did not result from an effect on locomotion or general feeding.

### M084 enhances brain-derived neurotrophic factor (BDNF)-tropomyosin-related kinase B (TrkB) signaling

**Fig 5:**
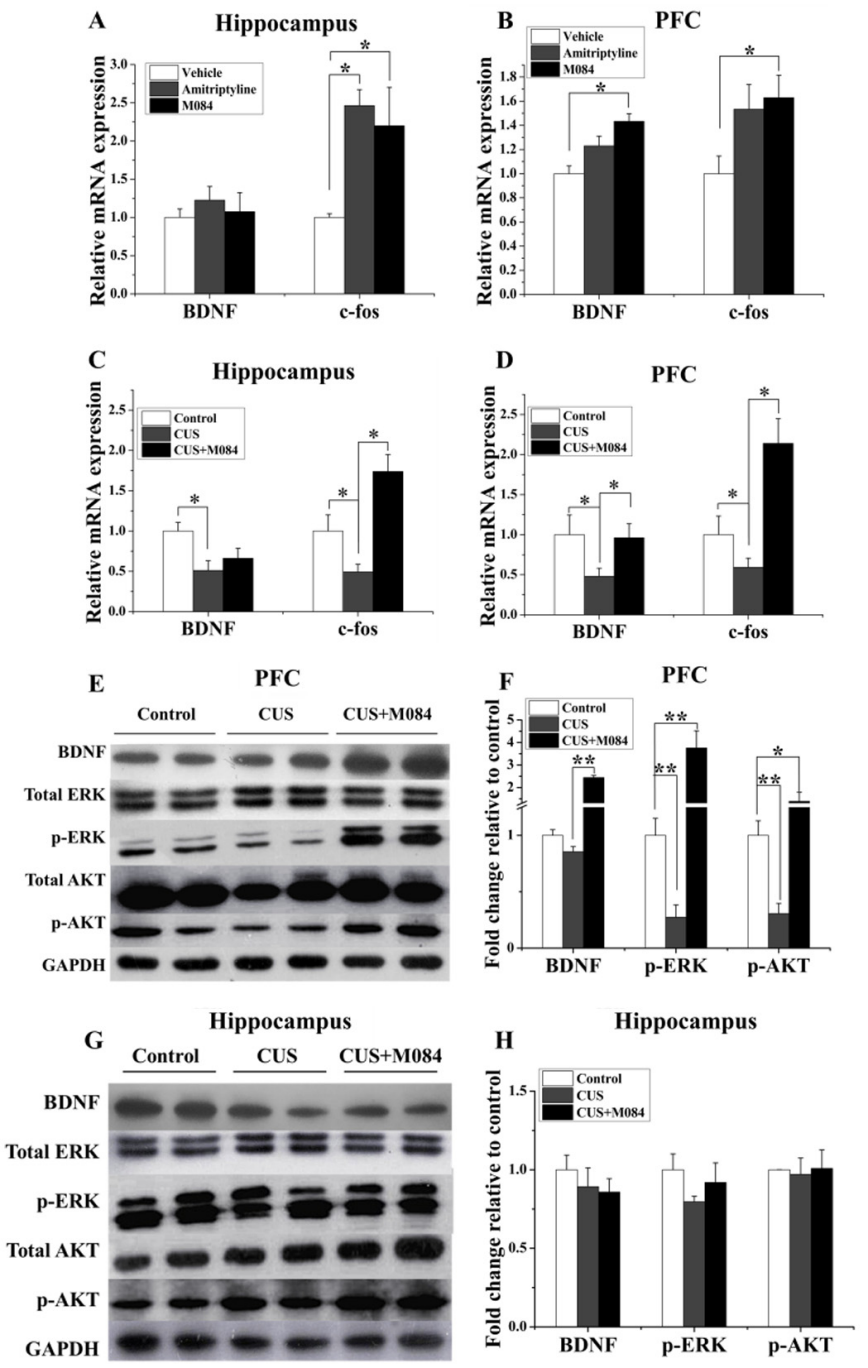
M084 treatment enhances BDNF-TrkB signaling in the prefrontal cortex (PFC). (A&B) mRNA levels of *BDNF* and *c-fos* in hippocampus (**A**) and PFC (B) of vehicle and M084-treated mice. Amitriptyline was used as a control for known antidepressant. (C&D) mRNA levels of *BDNF* and *c-fos* in hippocampus (**C**) and PFC (D) of vehicle and M084-treated mice subjected to CUS. Control animals were treated with vehicle only without CUS. (E&G) Representative western blots for mature BDNF, p-AKT (S473), total AKT, p-ERK1/2 (T202/204), and total ERK1/2 of PFC (E) and hippocampus (G). (F&H) The statistical results of protein levels of mature BDNF (normalized to that of GAPDH), p-AKT (S473)/total AKT, p-ERK1/2 (T202/204)/total ERK1/2 in PFC (F) and hippocampus (H). For all bar graphs, results are normalized to the control group with only the vehicle treatment and shown as mean ± SEM. N = 6/group,* *p* < 0.05, ** *p* < 0.01.

The BDNF signaling in neurons is decreased in depressed patients and animal models of depression [36]. Chronic, but not acute, treatment of antidepressants has been shown to increase BDNF levels in patients and animal models [36–38]. Improving BDNF signaling has been proposed to underlie the mechanism of antidepressants to remission [36]. Previous study also showed that antidepressants significant increased the *c-fos* expression in the prefrontal cortex (PFC) and hippocampus [39]. We found that an acute treatment of M084 increased the mRNA levels of *BDNF* and *c-fos* in PFC of normal mice [one-way ANOVA, *BDNF*: F (2, 15) = 8.394, *p* = 0.005; *c-fos*: F (2, 15) = 3.049, *p* = 0.082; Fig 5A]. M084 treatment also increased the mRNA level of *c-fos* [one-way ANOVA, F (2, 15) = 5.289, *p* = 0.021; Fig 5B], but not that of *BDNF* in hippocampi of normal mice [Fig 5B]. Importantly, the mRNA expression levels of both *BDNF* and *c-fos* were significantly decreased in PFC and hippocampi from mice subjected to CUS [Fig 5C and 5D]. The treatment of M084 strongly reversed the effect of CUS on *c-fos* expression in both hippocampus [one-way ANOVA, *BDNF*: F (2, 15) = 8.494, *p* = 0.003; *c-fos*: F (2, 15) = 11.838, *p* = 0.001; Fig 5C] and PFC [one-way ANOVA, *BDNF*: F (2, 15) = 5.740, *p* = 0.014; *c-fos*: F (2, 15) = 8.320, *p* = 0.004; Fig 5D] while it abolished the effect of CUS on *BDNF* expression in PFC but not hippocampus, suggesting a more prominent effect of M084 on *BDNF* expression in PFC than hippocampus, although the effect on *c-fos* expression is shared between the two brain regions. Consistent with the observed changes in the mRNA levels, the levels of mature BDNF protein in PFC, as shown by western blotting, were also increased by the treatment of M084 [one-way ANOVA, F (2, 15) = 11.432, *p* = 0.001; Fig 5E and 5F].

The downstream components of the BDNF signaling cascade, Ras–MAPK and PI3K-AKT, have been implicated in depression and treatment response [40]. Mice subjected to CUS also showed decreased levels of phospho-ERK and phospho-AKT in PFC, indicating that the downstream signaling of BDNF was compromised. The treatment of M084 led to marked increases in the phosphorylated levels of ERK and AKT without an obvious change in the total ERK and AKT expression, indicating improved BDNF signaling [one-way ANOVA, p-ERK: F (2, 15) = 40.984, *p* < 0.001; p-AKT: F (2, 15) = 9.958, *p* = 0.002; Fig 5E and 5F]. These results suggest that the antidepressant effect of M084 may involve increasing the level of BDNF and its downstream signaling.

## Discussion

TRPC channels are widely expressed in brain and involved in various aspects of brain function. Both TRPC4 and TRPC5 have been implicated in innate fear function, which represents a critical adaptive mechanism in response to environmental stress [22, 23]. In both TRPC4 and TRPC5 null mice, the deficits in innate fear were partly associated with diminished cholecystokinin (CCK) receptor signaling in amygdala neurons [22, 23]. Supporting the involvement of TRPC channels in CCK receptor signaling, a recent study showed that the CCK-induced increase in neuronal excitability required TRPC5 [41]. In line with the current finding that inhibiting TRPC4/C5 with M084 suppresses depression-like and anxiety-like behaviors in mice, stimulation of the CCK-B receptor produced anxiogenic-like and depressant-like effects while blockade of the CCK action promoted antidepressant-like and anxiolytic-like effects [42]. Thus, together with the literature data, our results suggest a novel pathway involving CCK receptors and TRPC4/C5 channels in depression/anxiety disorders.

We recently reported that the compound, M084, is a selective inhibitor of TRPC4/C5 [25]. In fluorescence Ca^2+^ assays, the compound exhibited an IC_50_ of 3.7 μM against TRPC4, while in fluorescence membrane potential assays, the IC_50_ values were 10.3 and 8.2 μM against TRPC4 and TRPC5, respectively [25]. Importantly, except for a very weak inhibitory effect on TRPC3 (IC_50_~50 μM), M084 showed neither agonistic nor antagonistic action on several other TRP channels, including TRPC6, TRPA1, TRPV1, TRPV3 and TRPM8 [25]. In addition, it did not affect the activities of native voltage-gated Na^+^, Ca^2+^, and K^+^ channels in mouse dorsal root ganglion neurons. The effectiveness of M084 on endogenous TRPC4-like activity was demonstrated by its blockade of the plateau potential mediated by TRPC4-containing channels in mouse lateral septal neurons [25]. Therefore, M084 represents an excellent pharmacological tool for investigation of physiological and pathological functions of native TRPC4 and TRPC5 channels. Here, we show that intraperitoneal injection of this novel TRPC4/C5 inhibitor produced anti-depressive and anti-anxiety effects in mice, further supporting its utility in pharmaceutical research. The way of drug administration used in the current study suggests that M084 can efficiently pass through the blood-brain barrier. Our acute toxicity assay also showed that mice receiving M084 at up to 400 mg/kg (body weight) survived well (data not shown). Therefore, M084 represents an excellent lead compound for further druggability investigations on neurological and psychiatric disorders.

To examine the anti-depressive and anti-anxiety effects of M084, we conducted multiple behavioral tests. As an important control, we showed that the administration of M084 did not affect locomotor activity of the mice. However, in both FST and TST, M084 caused significant reductions in the immobility time. This gave the first demonstration that M084 might be antidepressant, with no effect on the overall spontaneous locomotor activity. Since, anxiety is one of the most common mood disorders associated with depression [43] and several antidepressants, such as fluoxetine and escitalopram, have shown effectiveness in both depression and anxiety disorders [44, 45], we also tested the possible anxiolytic-like effects of M084 using EPM and L/D tests. In both cases, the administration of M084 increased the exploring activities in the open and illuminated areas, indicative of lowered anxiety. Our findings are in good agreement with the recent studies, which reported that gene ablation of either TRPC4 or TRPC5 decreased anxiety-like behaviors in mice [14].

To further investigate the anti-depression effects of M084, we used the CUS model, which is widely accepted as a mood related disorder animal model that captures core symptoms of depressive disorder [34]. The induction of CUS in mice led to decreased locomotor activity, increased immobility time in FST and prolonged latency to feed in a new environment, indicating the presence of depressed symptoms. The M084 treatment, although did not correct the moderately reduced locomotor activity, remarkably reversed the CUS-induced increase in immobility time in FST and reduced the latency to feed in NSFT. Previously, it was shown that the latency to feed in NSFT was shortened by chronic, but not acute, treatment of antidepressants [35]. Our finding that an acute (2 hrs) single treatment of M084 was beneficial in NSFT following CUS suggests that this new drug is fact acting, which is superior to existing antidepressants in mitigating depression caused by chronic stress. On the other hand, the acute treatment of M084 did not reverse anhedonia, which was also present in mice subjected to CUS (data not shown). The importance of the BDNF-AKT pathway in anti-depressive treatment has been well-established. Chronic treatment of antidepressants has been shown to increase the expression of BDNF in hippocampus and PFC [36, 38]. Genetic deletion or inhibition of BDNF actions is known to interfere with the antidepressant treatments [36, 46]. The fast onset antidepressant drug ketamine is thought to act on the BDNF-AKT pathway [47]. BDNF-TrkB signaling includes the activation of phosphatidylinositol-3 kinase (PI3K)-AKT (serine threonine kinase or protein kinase B) and Ras**-**mitogen-activated protein kinase (MAPK, also known as ERK for extracellular signal-regulated kinases) pathways [40]. It has been demonstrated that ERK signaling is reduced by stress and reversed by antidepressant and blockade of ERK produces depressive and anxiety behaviors [48, 49]. Moreover, AKT phosphorylation is decreased in PFC and hippocampus of depressed patients [50]. Our findings that CUS exposures decreased BDNF levels in mouse PFC and the treatment of M084 significantly reversed such a change in the CUS-exposed mice are entirely consistent with the current understanding about the contribution of the BDNF pathway in depressive disorders. They also agree with the report demonstrating TRPC5 as a regulator of neurogenesis [16]. In addition, we found that M084 treatment not only strongly enhanced the expression level of BDNF, but also the phosphorylation levels of AKT and ERK in PFC of CUS-exposed mice. These increases went beyond the levels reduced by the CUS induction, suggesting that TRPC4 and/or TRPC5 activities in PFC neurons are commonly associated with suppressing BDNF-AKT-ERK pathways under normal conditions and such suppression was further enhanced by the chronic stress imposed by the CUS paradigms.

The expression of c-fos is a marker of neuronal activity, and has been used to identify the regions of the brain and the neurotransmitter receptors that mediate the action of antidepressants [51]. Moreover, some fos-like positive neurons increased after antidepressant treatment in mood-related brain regions [51]. In the current study, M084 produced a significant increase of *c-fos* expression in the PFC both of normal mice and CUS mice, which is in line with previous findings [51].

Taken together, we demonstrate that the novel TRPC4/C5 inhibitor, M084, exerts rapid onset antidepressant-like and anxiolytic-like activities in mice under both normal and chronically stressed conditions. The action of M084 appears to include augmentation of the BDNF signaling pathway in PFC. These observations are in agreement with the current understanding that TRPC4 and/or TRPC5 channels play critical roles in brain function and suggest that these channels are linked to pathways responsible for the development of depressive and anxiety disorders. Currently, available treatment strategies for depression-related disorders centering on serotonergic and monoaminergic neurotransmitter mechanisms only work for a part of patient population and they only begin to exert their effects weeks following their administration. Although the possibility could not be ruled out that the effect of M084 was induced by targets other than TRPC4/5, such as other depression/anxiety-related receptors and transporters, our results propose M084 as a lead compound for further druggability research.

## Supporting Information

S1 ARRIVE Guidelines Checklist Completed “The ARRIVE Guidelines Checklist” for reporting animal data in the manuscript. (PDF)
